# Re‐exploring immune‐related side effects of docetaxel in an observational study: Blood hypereosinophilia

**DOI:** 10.1002/cam4.2062

**Published:** 2019-03-10

**Authors:** Diaddin Hamdan, Christophe Leboeuf, Christine Le Foll, Guilhem Bousquet, Anne Janin

**Affiliations:** ^1^ Medical Oncology Department Grand Hospital of East Francilien‐Marne‐la‐Vallée Jossigny France; ^2^ UMR_S1165, Inserm University of Paris‐Diderot Paris France; ^3^ University of Paris13 Villetaneuse France; ^4^ Medical Oncology Department Hospital Avicenne, APHP Bobigny France; ^5^ Pathology Laboratory Hospital St Louis, APHP Paris France

**Keywords:** allergic reaction, anticancer treatment, docetaxel, eosinophilia, hypersensitivity

## Abstract

Docetaxel is a major anticancer drug that can induce hypersensitivity reactions leading to deleterious treatment interruptions. Blood hypereosinophilia could be a biological sign, potentially lethal, of delayed visceral hypersensitivity reactions. We hypothesized this biological event is probably underreported. In this prospective observational study, we followed up 149 patients treated with docetaxel monotherapy for breast or lung cancer. For each patient, blood eosinophil counts were recorded during docetaxel treatment and up to 3 months after the end of docetaxel treatment. For all patients, blood eosinophil counts significantly increased under docetaxel chemotherapy (*P* < 0.01). Seven percent had persistent eosinophilia after the end of treatment. Four patients had blood eosinophil counts over 1000/mm^3^ with severe cardiac, cutaneous and digestive toxicities, and docetaxel imputability was confirmed using drug‐imputability scales. For two of these four patients, tissue biopsies were performed during the time of hypereosinophilia and of severe toxicities. Specific immunostainings and electron microscopy found numerous degranulating mast cells and eosinophils. Our study demonstrated that eosinophilia is frequent under docetaxel and could lead to severe complications, implicating eosinophils and mast cells, and possibly IgE. One way of treating hypersensitivity reactions could be by targeting IgEs with omalizumab, an anti‐IgE monoclonal antibody approved for the treatment of severe allergic asthma, and successfully used in food and poison‐induced anaphylactic reactions.

## 1 INTRODUCTION

Docetaxel, a semi‐synthetic taxane inhibiting microtubule depolymerization, is approved for breast and lung cancer treatment. It is frequently responsible for drug‐induced hypersensitivity reactions in up to 50% of patients,^1^, ^2^ thus leading to deleterious treatment interruptions. Rapid drug desensitization protocols are effective in the management of nonsevere hypersensitivity reactions, thus limiting treatment interruptions.^3^, ^4^, ^5^, ^6^, ^7^ However, severe delayed visceral hypersensitivity reactions, potentially lethal from visceral complications, are excluded from desensitization protocols.

We recently reported a case of docetaxel‐induced blood hypereosinophilia with a severe digestive allergic reaction.^8^ We hypothesized that drug‐induced blood eosinophilia, probably underreported, could be a biological sign of hypersensitivity reaction, and could also predict severe delayed visceral hypersensitivity reactions.

In this observational study, we aimed to determine the incidence of docetaxel‐induced eosinophilia, and whether it could be an early biological event predictive for the risk of delayed visceral hypersensitivity reactions.

## 2 MATERIALS AND METHODS

### 2.1 Inclusion criteria, clinical, and biological data

This study was approved by our local Institutional Review Board ‐IRB 00006477.

One hundred and forty‐nine patients were included over a period of 1 year. All of them were being treated with docetaxel monotherapy for breast or lung cancer, as specified in the inclusion criteria (Table 1).

**Table 1:** Inclusion criteria

Inclusion criteria	Exclusion criteria
Breast or lung cancers	Cancers of other origins
Localized or metastatic cancer	
Docetaxel monotherapy	Docetaxel combination therapy
Available blood analyses before, during and after docetaxel treatment	Blood analyses not available during docetaxel treatment

**Figure 1:**
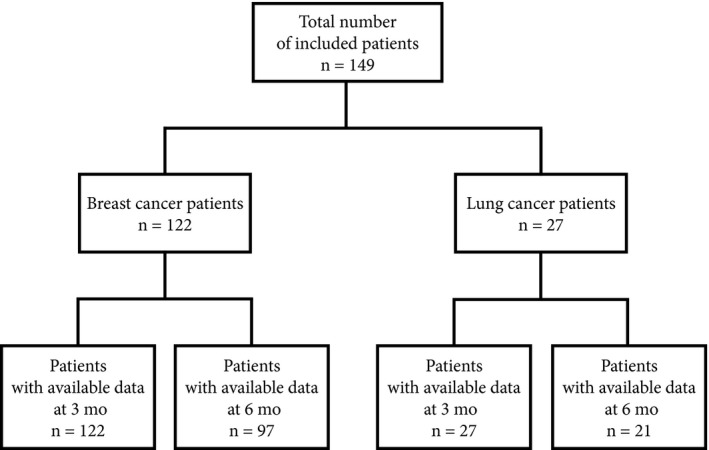
A flow‐diagram to show the number of patients included in the study and those with unavailable data

For each patient, blood eosinophil counts were recorded at the beginning of docetaxel treatment, before each cycle, and up to 3 months after the end of docetaxel treatment when data were available. At this last time‐point, data were available for 79% of the patients (Figure 1). For the whole population, the blood eosinophil count was retrieved at 1 week before the beginning of docetaxel, at the end, and at 3 months after the end of docetaxel treatment.

For each patient, we considered that the increase in blood eosinophil counts was significant when it was at least twice as high compared to the count before initiation of docetaxel treatment.

For patients with blood eosinophil count >1000/mm^3^, various tests were conducted to eliminate other possible causes of eosinophilia (Table S1).

For pharmacological imputability of docetaxel, we calculated an imputability score using the French and the North American validated pharmacovigilance scales.^9^, ^10^

For each patient, docetaxel‐induced hypersensitivity reactions of any type (wheal‐and‐flare reactions, maculopapular eruptions, urticaria, itching, angioedema, local edema, bronchospasm, gastrointestinal symptoms, anaphylaxis, etc) were recorded and graded according to CTCAE‐NCI grading scale version 5. Immediate and delayed hypersensitivity reactions were considered, and delayed hypersensitivity reactions occurred at least 6 hr after each administration of docetaxel.^11^

### 2.2 Tissue analyses and characterization of eosinophils and mast cells

Among the four patients with blood hypereosinophilia >1000/mm^3^, we were able to perform tissue analyses for two patients with eosinophil counts for whom tissue samples were obtained at the time of blood eosinophilia.

We used anti‐tryptase and anti‐eosinophil peroxidase (EPO) antibodies to differentiate and count mast cells and eosinophils. These two immunostainings were performed on 5 μm‐thick tissue sections using indirect immunoperoxidase staining, with rabbit polyclonal anti‐human EPO (ab104530, Abcam, Cambridge, UK) and monoclonal mouse anti‐human tryptase (clone G3, Santacruz, Heidelberg, Germany) as primary antibodies. Controls included omitting the primary antibody and using an irrelevant antibody of identical isotype. The analysis focused on the number and distribution of mast cells and eosinophils in the different tissue sections. Tissue sections were analyzed using an Olympus AX 70 microscope with a 0.344‐mm^2^ field size at 400× magnifications (Olympus, Tokyo, Japan). Images were systematically taken using SAS software for each immunostaining image.

For the ultrastructural analysis, tissue samples were fixed in 2% glutaraldehyde‐buffered 0.1 M. cacodylate and embedded in epoxy resin. Ultra‐thin sections were stained with uranyl acetate and lead citrate and analyzed using a Hitachi‐7650. The images of the distribution of mast cells and eosinophils and their state of degranulation were captured.

### 2.3 Statistical analysis

Categorical variables were summarized as the number (percentage) and continuous variables were summarized as the mean or the median.

A comparison of the median value of the three matched‐sample of blood eosinophil count was performed (ie, pretreatment period, at the end, and at 3 months after the end of docetaxel treatment) using the Wilcoxon's test.

All tests were two‐sided and the threshold for statistical significance was set to *P* < 0.05. The data were analyzed using the BiostaTGV site (http:\\marne.u707.jussieu.fr/biostatgv, accessed in Avril 2018).

## 3 RESULTS

### 3.1 Patients

Patients were recruited from January 2017 to December 2017, and inclusion criteria are detailed in Table 1. A total of 149 patients with breast or lung cancers treated with docetaxel monotherapy were included during this period. Their characteristics are detailed in Table 2:81% had breast cancer, with a median age of 55 and 61 years for breast and lung cancer patients, respectively.

**Table 2:** Patients’ characteristic

Patients		Breast	Lung	Whole cohort	At least twofold increase in blood eosinophil count during follow‐up period
Number (%)		122 (81)	27 (18)	149 (**100**)	73 (**49**)
Mean age (years)		55	61	58	60
Allergic history (%)		18 (13)	2 (7)	20 (13)	9 (12)
Mean number of docetaxel cycles		2.89	4.63	3.76	2.58
Mean dose of cycle 1 (mg)		160.72	123	141.86	151.46
HSRs other than blood eosinophilia	G1‐2^a^ (%)	42 (34)	8 (29)	50 (**33**)	30 (**41**)
	G3‐4^a^ (%)	9 (7)	2 (7)	11 (**7**)	7 (**9**)

Bold values are corresponding to percentages.

HSR: hypersensitivity reactions.

a G: Grade according to Common Terminology Criteria for Adverse events of the United States National Cancer Institute, CTCAE‐NCI v.5.0.

### 3.2 Blood eosinophil counts under docetaxel chemotherapy

**Figure 2:**
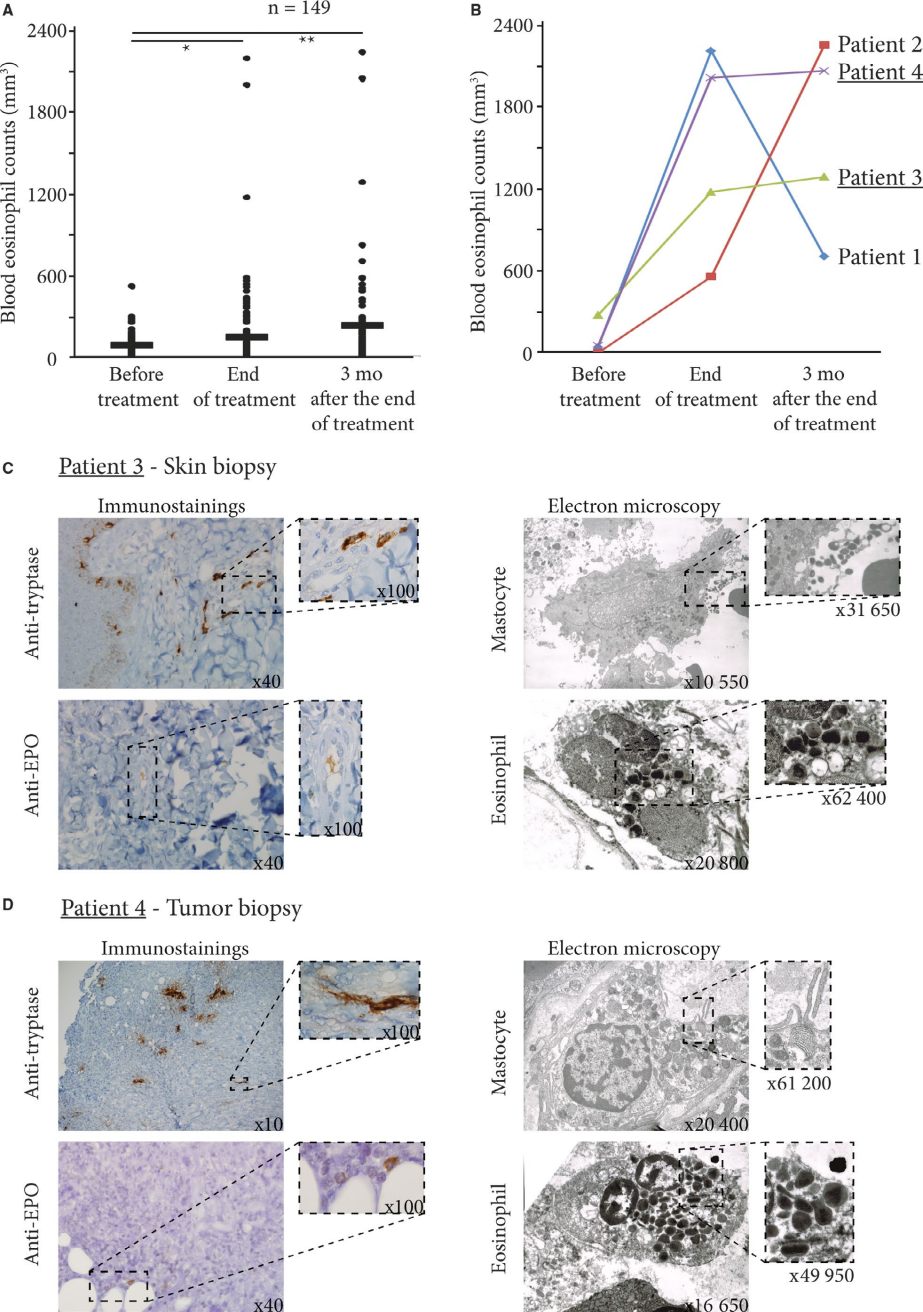
(A) Blood eosinophil count curves in the course of docetaxel treatment and up to 3 months after discontinuation for all patients included in the study. (B) Blood eosinophil count curves for the four patients who developed blood eosinophilia in the course of docetaxel treatment. (C) Skin biopsy for Patient 3 who had persistent blood eosinophilia well beyond 6 months after the end of docetaxel treatment, accompanied by severe chronic pruritus. Anti‐eosinophil peroxidase and anti‐tryptase immunostainings show eosinophils and mast cells infiltrating the deep dermis. This was confirmed by electron microscopy showing many degranulating eosinophils and mast cells. (D) Tumor sample for Patient 4 who had hypereosinophilia at the time of breast surgery. Anti‐eosinophil peroxidase and anti‐tryptase immunostainings also show eosinophils and mast cells infiltrating the tumor. This was confirmed by electron microscopy which evidenced numerous degranulating eosinophils and mast cells

We have compared the median of blood eosinophil counts before, at the end and three months after the end of docetaxel treatment. Among the 149 patients, 73 (49%) had at least a twofold increase in their blood eosinophil counts during the follow‐up period (Table 2). We have compared the median of blood eosinophil counts before, at the end and 3 months after the end of docetaxel treatment. Median blood eosinophil counts significantly increased under docetaxel chemotherapy, from 77/mm^3^ before treatment, to 135/mm^3^ and 221/mm^3^ at 3 and 6 months respectively (*P* < 0.01) after docetaxel initiation (Figure 2A). Three months after the last cycle of docetaxel, blood eosinophil counts remained higher than 500/mm^3^ in 7% of the patients (Figure 2A and Figure S1), with comparable results in the two cancer types (Figures S2 and S3).

### 3.3 Docetaxel‐induced hypersensitivity reactions

When we looked at all‐grade docetaxel‐induced hypersensitivity reactions other than blood eosinophilia, they occurred for 66 of the 149 patients (40%) (Table 2). Interestingly, among the 52 patients with a significant increase in blood eosinophil counts, 50% had a hypersensitivity reaction manifestation other than blood eosinophilia (Table 2). Table 3 reports the different types of hypersensitivity reactions, and shows that all‐grade hypersensitivity reactions occurred in 21 patients (14%), leading to a premature discontinuation of planned docetaxel treatment.

**Table 3:** Types of hypersensitivity reactions (HSRs)

HSR events	Delayed				Immediate				At least twofold increase in blood eosinophil count during follow‐up period	
	Visceral		Skin		Visceral		Skin			
	G1‐2^a^	G3‐4^a^	G1‐2^a^	G3‐4^a^	G1‐2^a^	G3‐4^a^	G1‐2^a^	G3‐4^a^	<500/mm^3^	>500/mm^3^
Number	28	2	24	5	8	6	4	1	51	12
Median time to onset (day)	42	30	30	21	1 hr	1 hr	1 hr	1 hr	76	65
Median duration	1 week	7 months	1 week	2 week	1 hr	2 hr	1 hr	1 hr	9 months	7 months
Docetaxel stopped	2	1	3	4	4	6	1	—	6	1

HSR: hypersensitivity reactions; BEC: blood eosinophil count; N: blood eosinophil count at the initiation of docetaxel treatment.

^a^ G: Grade according to Common Terminology Criteria for Adverse events of the United States National Cancer Institute, CTCAE‐NCI v.5.0.

Four patients (2.6%) had blood eosinophil counts over 1000/mm^3 ^(Figure 2B). We eliminated other possible etiologies of eosinophilia and thus confirmed the imputability of docetaxel using drug‐imputability scales (Table 4). Patient 1 had an NCI‐CTCAE‐v5 grade II diarrhea without severe complications; Patient 2 had an NCI‐CTCAE‐v5 grade III cardiac flutter at the end of the docetaxel treatment despite the absence of any cardiac risk factor. For Patient 3, blood eosinophilia persisted well beyond 6 months after the discontinuation of docetaxel, with severe chronic pruritus justifying a skin biopsy. Patient 4 received docetaxel and had hypereosinophilia at the time of breast surgery.

**Table 4:** Drug imputability scores for the four patients with blood eosinophil counts over 1000/mm3

	Drugs	Adverse drug reaction probability scale^9^	French imputability score^10^			
		Score	IS	C	S	Intrinsic imputability
Patient 1	**Docetaxel**	**6**	**2**	**3**	**3**	**I6**
	Ondansetrone	0	2	1	2	I2
	Prednisone	0	2	1	2	I2
	Metoclopramide	0	2	1	2	I2
	Paracetamol	−2	2	1	2	I2
	Loperamide	−2	2	0	2	I0
	Lansoprazole	−2	2	0	2	I0
	Phloroglucinol	−2	2	0	2	I0
	Diosmectite	−2	2	0	2	I0
Patient 2	**Docetaxel**	**6**	**2**	**3**	**3**	**I6**
	Ondansetrone	0	2	1	2	I2
	Prednisone	0	2	1	2	I2
	Metoclopramide	0	2	0	2	I0
	Esomeprazole	−2	2	0	2	I0
	Hydroxyzine	−2	2	0	2	I0
	Sotalol	−2	2	0	2	I0
	Nicopatch	−2	2	0	2	I0
	Levetiracetam	−2	2	0	2	I0
	Clobazam	−2	2	0	2	I0
Patient 3	**Docetaxel**	**6**	**2**	**3**	**3**	**I6**
	Ondansetrone	0	2	1	2	I2
	Prednisone	0	2	1	2	I2
	Metoclopramide	0	2	1	2	I2
Patient 4	**Docetaxel**	**6**	**2**	**3**	**3**	**I6**
	Ondansetrone	0	2	1	2	I2
	Prednisone	0	2	1	2	I2
	Metoclopramide	0	2	1	2	I2
	Omeprazole	−2	2	1	2	I2

Bold values are corresponding to calculated scores according to each pharmacological scales.

IS: Informativeness score, C: chronology, S: semiology

On the skin of Patient 3 and the breast cancer of Patient 4, specific immunostainings (anti‐eosinophil peroxidase, anti‐tryptase) and electron microscopy found numerous degranulating mast cells and eosinophils; for Patient 4, we found clustered tryptase‐expressing mast cells at the invasive front of the tumor (Figure 2B,C).

### 3.4 Literature review

For the literature review of hypereosinophilia cases imputable to anticancer drugs, we used an ad‐hoc algorithm composed of both thesaurus and free text terms to search the Medline database up to 2 May 2018. We used the following algorithm: ("Eosinophilia"[Mesh] OR "Eosinophilia" OR “eosinophilic” OR “eosinophilic syndrome” OR “hypereosinophilia”) AND ("Neoplasms"[Mesh] OR “cancer”) AND ("Drug Hypersensitivity Syndrome"[Mesh] OR "Antineoplastic Agents"[Mesh] OR "Drug Therapy"[Mesh] OR “chemotherapy” OR "drug‐induced"). With the limits: Species = human and blood eosinophil count >1500/mm^3^, 818 articles were initially identified. We screened the papers retrieved initially on title and abstract, and finally on full text. Twenty‐three publications on hypereosinophilia imputable to an anticancer agent were identified, 19 were case reports, two others were phase I clinical trials, one was an observational cohort, and the last was a literature review (Table 5).

**Table 5:** Chemotherapy‐induced hypereosinophilia publications

Name/class of drug	Type	References
Check‐point inhibitor	Review	Melanoma Res. 2017 Jun;27(3):271‐273
Pemetrexed	CR	Am J Dermatopathol. 2017 Jan;39(1):e1‐e2
Vismodegib	CR	Australas J Dermatol. 2017 Feb;58(1):69‐70
Anti‐PD1	CR	Eur J Cancer. 2017 Aug;81:135‐137
Pembrolizumab	CR	Cancer Immunol Res. 2016 Mar;4(3):175‐8
Vemurafinib	CR	Dermatology. 2016;232(1):126‐8
Pan‐class I PI3K inhibitor	Phase I	Oncologist 2015; 20(3): 245‐46
Cisplatin	CR	Case Rep Pulmonol. 2014;2014:209732
Ipilumumab/anti‐CTLA4	Multi‐center cohort	PLoS One 2013; 8(1): e53745
Lenalidomide	CR	Rinsho Ketsueki. 2016;57(12):2502‐2506
Lenalidomide	CR	Eur J Dermatol 2012;22(6):799‐800
Tosedostat/aminopeptidase inhibitor + paclitaxel	Phase I	Br J Cancer 2010;103(9): 1362‐68
Chlorambucil	CR	Pharmacology 2009;83:148‐149
Imatinib/dasatinib	CR	Ann Allergy Asthma Immunol. 2017;119(1):85‐86
Imatinib	CR	Ann DermatolVenereol 2008;135(5):393‐6
	CR	Ann DermatolVenereol 2006;133:686‐8
	CR	Lancet Oncol 2005;6(9):728‐9
Dacarbazine	CR	Ann DermatolVenereol 2006;133(2):157‐60
Fludarabine	CR	Ann Hematol 2002;81(5):292‐3.
	CR	Ann Hematol 1999;78(10):475‐7
13‐cis‐retinoic acid	CR	Med PediatrOncol 1999;32(4):308‐10
Tegafur	CR	J Gastroenterol 1994;29(1):88‐92
Bleomycine	CR	Chest. 1985 Jul;88(1):103‐6

CR: Case report

## 4 DISCUSSION

Our study confirmed our hypothesis that docetaxel‐induced blood eosinophilia is largely underestimated since a twofold increase in blood eosinophil count occurred in half of the treated population. It was frequently associated with other clinical manifestations of immediate and delayed hypersensitivity reactions, supporting our hypothesis that blood eosinophilia is a biological sign of hypersensitivity reaction. In addition, it was severe, over 500/mm3, and durable over time for 7% of the patients, comparable to the 7% of patients with grade 3‐4 hypersensitivity reactions in phase I clinical trials with docetaxel.^12^, ^13^ It led to visceral complications for four of the 149 patients, and in all four cases the increase in blood eosinophil count preceded the visceral complication. Blood eosinophilia could be thus an early biological sign predictive for the risk of docetaxel‐induced delayed visceral hypersensitivity reactions.

Strikingly, eosinophilia is not reported in clinical trials using docetaxel, possibly because of corticoid premedication which limits the increase in blood eosinophil counts, and also because blood eosinophilia can occur after the end of docetaxel treatment when systematic blood counts are no longer performed. Even for other drugs, drug‐induced blood eosinophilia is rarely reported, as our literature review shows.

In case of drug‐induced blood eosinophilia, a desensitization protocol, similar to those implemented for immediate hypersensitivity reactions,^3^, ^4^, ^5^, ^6^, ^7^ might be useful to avoid delayed visceral complications.

One limitation to our observational study is the limited number of patients and tissue samples analyzed. Despite this, we were able to demonstrate that docetaxel‐induced eosinophilia is a frequent biological sign of hypersensitivity reaction that can predict delayed visceral complications. In addition, with only two biopsy samples obtained at the time of hypereosinophilia with visceral complications, we confirmed the tissue infiltration by degranulating eosinophils and mast cells, as reported in our previous publication.^8^

Docetaxel‐induced hypersensitivity reaction is an inflammatory reaction resulting from the activation of eosinophils and mast cells,^2^ themselves able to enhance their own recruitment and activation through an autocrine loop.^14^ IL‐5 and IL‐13 auto‐secretion boost IgEs and also induce eosinophil activation and degranulation through their low‐affinity IgE receptor. One way of treating hypersensitivity reactions could be by targeting IgEs with omalizumab, an anti‐IgE monoclonal antibody approved for the treatment of severe allergic asthma, and successfully used in food and poison‐induced anaphylactic reactions.^15^

In conclusion, our observational study demonstrated that docetaxel‐induced blood eosinophilia is a frequent early biological sign of hypersensitivity reaction that can predict delayed visceral complication.

## CONFLICT OF INTEREST

The authors have declared no conflicts of interest.

## ETHICS

This work was approved by the local Institutional Review Board ‐IRB 00006477 under the number: 16‐053. It was also approved by the National Committee on private freedoms (CNIL) under the number: 1988828 v 0.

Informed consent from each patient was obtained prior to inclusion in the study.
