# Supplementary material for: Re‐exploring immune‐related side effects of docetaxel in an observational study: Blood hypereosinophilia
Source: Cancer Med. 2019 Mar 10;8(5):2005–12. doi: 10.1002/cam4.2062 (PMC6537007; doi:10.1002/cam4.2062)
Supplement: Supplementary file 4 [file CAM4-8-2005-s004.docx]

Supplementary Table S 1: Laboratory analysis realized to eliminate possible causes of blood eosinophilia

| **Laboratory analyses** |
| --- |
| **Parasitology** |
| - Stool analyses (3 tests) - Serology: toxocariasis, bilharziasis, distomatosis schistosomiasis, - Search in biopsy for cryptosporidium and microsporidium |
| **Immunology** |
| - IgE dosage - Complement dosage - ANCA, extractable nuclear antigen antibodies, anti-nuclear antibodies |
| **Hematology** |
| - Clonal T cell in the blood - FIP1L1-PDGFR rearrangement |
| **Bacteriology and virology** |
| - Salmonella, klebsiella oxytoca, shigella, clostridium difficile, campylobacter, yersinia enterocolitica - Cytomegalovirus, Epstein-Bar, herpes simplex 1, herpes simples 2, varicella zoster , adenovirus, enterovirus, human herpes virus 6A, 6B and 8 |

ANCA: anti-neutrophil cytoplasmic antibodies. Q-PCR: quantitative polymerase chain reaction
